# Supplementary material for: Are carbon emissions trading and green financial instruments synergistic? -Comprehensive quantitative research based on content analysis
Source: PLoS One. 2024 Mar 7;19(3):e0298601. doi: 10.1371/journal.pone.0298601 (PMC10919601; doi:10.1371/journal.pone.0298601)
Supplement: S1 Data — (ZIP) [file pone.0298601.s001.zip › Data/地级市Z_F工作报告.zip!/╣π╢1⁄2/╜¡├┼╩╨/└┤╫╘╣1⁄2╓┌║┼í╛┬φ┐╦╩2╛▌═°í┐_2004.docx]

各位代表：

　　我代表市人民政府向大会作政府工作报告，请予审议，并请政协委员和其他列席人员提出意见。

２００３年工作回顾

　　２００３年是不平凡的一年。全市人民经受住非典和强台风等严峻考验，团结一致，锐意进取，三个文明建设有新发展，荣获“国家卫生城市”、“中国人居环境范例奖”和“全国双拥模范城”三连冠，较好地完成了市十二届人大一次会议确定的各项目标任务。

　　一、经济发展速度质量效益同步提高

　　主要经济指标全面增长。全市生产总值完成７３１．１亿元，增长（与上年同比，下同）１１．１％，三大产业结构比例由上年的１１：４８：４１调整为１０：４９：４１，人均ＧＤＰ达１９１５７元，增长１０．５％；地方财政一般预算收入完成２９．４亿元，增长１６．３％，增幅自１９９４年以来首次超过全省平均水平；全社会固定资产投资完成１６１．１亿元，增长１７．７％；外商直接投资８．５亿美元，增长１５．５％外贸出口总额３６．５亿美元，增长２３．８％；社会消费品零售总额实现２７７．８亿元，增长１２．０％；各项主要经济指标全面完成了预定目标。

　　工业主导地位进一步增强。全社会工业总产值实现１５３９亿元，增长１６．２％，是７年来增速最快的一年。重工业增速首次超过轻工业。机电、纺织化纤、造纸、食品等产业快速增长。产值超１０亿元企业达到１１家，大长江、开平涤纶跻身中国企业５００强。当年先后获得“中国摩托车产业示范基地”、“中国（新会）不锈钢制品生产基地”和“中国五金卫浴产业基地”等称号。大长江摩托车产销量双超１００万辆，跃居全国摩托车行业的龙头。新增“李锦记”、“味事达”等一批全国、全省名牌产品。新增高新技术企业２８家，高新技术产品产值２９７．２亿元，增长２２％，占全市工业总产值１９．３％，比上年提高５个百分点。工业经济效益明显提高，规模以上企业经济效益综合指数达１３２，同比提高１０个百分点。

　　农业和农村经济加快发展。农业结构进一步优化，粮经作物比例调整为４８：５２。农业产业化程度进一步提高，龙头企业带动农户１９．８万户，增长３１．３％。现代农业示范区建设有新发展，８９个农业生产基地通过了省级无公害农产品认证，１２个产品获得国家级绿色食品认证。镇级经济发展较快，镇级财政收入增长２１．４％，财政收入超千万元的镇（办事处）达４０个，比上年增加８个。农村税费改革成效显著，全年农民减负２．９亿元，农民人均负担从１４４元下降到２６．７元，减负幅度达８１．５％。

　　第三产业稳步发展。成功举办了２００３中国（江门）侨乡旅游节，恩平市被命名为“中国温泉之乡”。受非典影响较大的交通运输、旅游、餐饮等行业全年发展比预期的好。汽车、住房、通讯、旅游、医疗保健、文化教育和体育健身等消费需求增长较快。第三产业增加值完成３０１．９亿元，增长１１．５％。

　　二、内外源经济持续快速增长

　　民营经济发展势头良好。制定实施了《关于加快民营经济发展的若干意见》及配套政策，为民营经济营造了宽松的发展环境。全年新增私营企业３０９６户，个体工商户２．５万户，分别增长２２．２％和１０．７％；私营企业新增注册资金３０．３亿元，增长５０．６％，其中注册资金超千万元的有２９户，超亿元的４户。规模以上民营工业企业实现总产值３２０亿元，增长２８．２％。个体和私营经济实现税收１３．１亿元，增长２４．６％。新增有进出口经营权的民营企业１４７家，民营企业出口总额达６．７亿美元，增长１．５倍。

　　外向型经济再创新高。举办了电子信息业合作交流会、机电产品博览会、新会五金不锈钢产品展销会以及开平牛仔服装节和卫浴展等招商活动。新批合同利用外资１１．０亿美元。引进了亚太纸业等２９个超千万美元的外资项目。新引进了世界５００强之一的泰国正大集团。中油ＢＰ江门石油有限公司、江门联和发动机、开平中晖纤母粒、新会中集、大长江等５家企业跻身全国外商投资企业５００强。外贸出口上新台阶，出口超千万美元的企业达７９家。机电产品和高新技术产品出口分别增长３１％和５１％。

　　改革攻坚取得重大突破。市直国企完成改制４７家，累计改制１８８户，完成目标任务的８８．７％。完成了美雅集团和恩平广联泰等一批重大项目的产权转让和重组。在加大改革力度的同时，妥善处理改革与稳定的关系，做好职工安置等各项配套工作。稳步推进国有粮食企业改革。深化公有资产管理体制改革，成立了市资产管理局。公共财政体系建设有新发展，理顺了市区财政管理体制，对市直纳入规范公务员收入的单位实施财政国库集中支付管理。

　　三、基础设施建设和城乡建设再创佳绩

　　重点工程建设取得新突破。加快推进４８项重点工程建设。亚洲规模最大的火力发电厂台山电厂１号机组（６０万千瓦）顺利并网发电；开阳高速公路通车，我市高速公路通车里程达３０３公里，居全省地级以上市首位；江中、江鹤二期、江珠高速公路建设及江肇高速公路、广珠轻轨江门支线工程前期工作进展顺利。

　　城乡建设又有新变化。围绕创建国家卫生城市，开展了一系列城市美化、绿化、亮化工程，２６项江门市区城建重点项目建设进展顺利。金汇、金凯悦、丽宫等五星级酒店及五邑华侨广场会展中心等重点项目加紧建设。滨江大道建设拉开序幕。在世界银行发布的《中国投资环境报告》中，我市被列为Ａ类地区，位居全国２３个大中城市的前列。城镇化建设继续推进，新撤并３个镇，２０００年以来累计撤并２２个镇，撤并率达２３．９％。完成了５个中心镇的规划修编。水环境综合整治初见成效，潭江水质保持较好水平。市区全年空气质量日报优良率达９８．９％，大气环境质量达到国家二级标准。生态环境明显改善，全市２４３万亩生态公益林保护良好，建成８个省级生态示范园区。水利基础设施建设进一步加强，完成江新联围睦洲堤段、锦江水库等１６项加固工程以及１０４宗小型水库除险加固工程。

　　四、社会各项事业全面进步

　　公共卫生建设有序展开。我市坚决贯彻党中央、国务院和省委、省政府的各项部署，沉着应对，靠前指挥，精心组织，措施果断，取得了抗击非典的阶段性胜利。建立起公共卫生突发事件预警和应急机制，制定了防治非典应急工作预案。健全疾病预防控制、医疗救治、卫生监督三大体系，建立疫情信息网络系统，增强了政府应对公共卫生突发事件的能力。疾病预防控制中心和市人民医院功能大楼等卫生基础设施建设进展良好。农村公共卫生设施不断完善，新型农村合作医疗人口覆盖率达３５．１％。

　　文化名市建设全面启动。《江门市建设文化名市规划纲要》起步实施。华侨博物馆、电视中心、体育公园、城市雕塑等一批文化设施建设启动。聘任了２１位五邑籍院士为我市科技顾问，举行了“星光大道”命名仪式。粤剧《再打金枝》获得全国“映山红”戏剧节最高奖项。

　　科技教育事业涌现一批新亮点。创建了“国家火炬计划江门新材料产业基地”，新增３个省级技术创新专业镇，建立省、市级工程技术研发中心３０家。全市专利申请量达１８４８件，同比增长６８％。信息化综合指数达５５％，比上年提高５个百分点。教育事业蓬勃发展。五邑大学招生２５２１人，新增４个硕士点。高考录取率达８７．２％，高于全省平均水平５个百分点。适龄青年高等教育毛入学率２４．１％，高于全省平均水平６．６个百分点。创建省一级中小学４所，地市一级中小学１７所，增加地市一级以上中小学优质学位２．５５万个。完成了９６所中小学布局调整工作。

　　其他各项事业取得新进展。社区建设进一步加强，推动了社区内单位文化体育设施向社会开放，建成“星光老年人之家”１５３个。体育事业有新发展，江门五邑籍运动员在世界、国家级赛事上获得好成绩，举办了一批具有影响力的国际国内赛事。新闻、出版、档案、民政、宗教、人防、残联、老龄、气象等各项事业有新进步。外事、侨务、台湾事务工作取得新成绩。

　　人民生活水平进一步提高。全市在岗职工年人均工资１２４０５元，增长１１．１％。农村居民年人均纯收入４９６０元，增长３．５％。城乡居民储蓄存款余额增长１５．４％。扶贫工作取得新成效，全市集体年收入３万元以下的３８５条贫困村，已有１１６条村实现了脱贫。计划生育工作巩固提高，人口出生率下降了０．２３个千分点，自然增长率降至３．０４‰，全面完成了省下达的人口控制和计划生育指标。妇女儿童合法权益得到保障，妇幼保健工作得到进一步加强。

　　社会保障体系不断完善。社会保险覆盖面不断扩大，企业养老、失业、医疗参保人数分别达４２．５万人、３７．０万人和３８．８万人，参保率分别为９６．６％、９７．９％和１０５．２％。全面建立了镇（办事处）、社区劳动保障工作机构。全市新增２．７万个就业岗位，１．１万名下岗失业人员实现再就业，城镇登记失业率为３．２％，就业形势稳定。全市享受定恤定补重点优抚对象６４３２人，全年发放定恤定补资金１５９１万元；全市城乡最低生活保障对象４．９万人，全年发放低保救济金２５００万元。

　　社会治安综合治理取得明显成效。深入开展严打整治斗争，重点打击“双抢”、盗抢机动车等犯罪活动。社会治安防控网络进一步完善。“禁毒０３工程”取得初步成效。健全信访工作制度，落实信访责任制，妥善处理人民内部矛盾，维护了社会大局稳定。

　　五、政府自身建设有所增强

　　民主法制建设进一步推进。各级政府自觉接受人大及其常委会的监督，坚持重大问题与人民政协协商。建立和拓宽了政府与市民沟通渠道，广泛听取各方面意见。全年办理人大代表建议１５４件，政协提案１３９件。建立起全市办理人大、政协提案建议工作网络。全面推行政务、厂务、镇务、村务公开。普法宣传教育更为广泛，开展“民主法治示范村”建设，村民法律意识进一步增强。加强行政执法队伍建设和行政执法监督。法律服务、监狱、劳教工作得到加强。统计、审计和监察工作水平不断提高。

　　政务管理和机关作风建设有创新。颁布了《江门市人民政府工作规则》。以市政府办公室为试点，导入ＩＳＯ管理体系，创新机关管理体制，形成新的绩效评价体系。实行政府目标管理，制定考核目标１７５项，完成１７１项。进一步转变政府职能，推进行政审批制度改革。大力推行电子政务。建设部试点的１２３１９城建服务热线取得良好效果。政府廉政建设和反腐败工作扎实推进。完善政府采购制度，进一步落实“收支两条线”管理工作。

　　各位代表，回顾一年的工作，我们的成绩来之不易。这是贯彻落实党的十六大精神和“三个代表”重要思想的结果，是省委、省政府和市委正确领导的结果，是各级人大和政协监督支持的结果，是全市人民和中央、省驻我市各单位及驻军部队团结奋斗的结果，是各民主党派、社会各界人士以及广大外来投资者和建设者、港澳台同胞、海外侨胞关心支持的结果。在此，我代表市人民政府，向关心支持江门经济社会发展的同志们、朋友们，向港澳台同胞和海外乡亲表示崇高的敬意和衷心的感谢！

　　我们清醒地看到，过去一年政府工作仍存在许多薄弱环节和问题，主要是：经济整体素质和竞争力比先进地区有明显差距；县域、镇域经济发展不均衡；城乡发展矛盾扩大；人民生活水平提高不够快；政府服务在不少方面不尽人意；安全生产措施还不够落实；等等。对这些问题，市政府正高度重视，科学谋划，加大解决力度。

２００４年工作任务

　　２００４年是实现“十五”计划目标的关键一年。作为珠三角后发地区，我们既要正视与珠三角先进地区差距拉大的严峻形势，增强加快发展的紧迫感和危机感；又要树立“后发争先”的强烈意识，增强加快发展的信心和决心。从国际国内环境以及我市近年来的发展变化看，江门正进入新的大发展机遇期，具备了后发争先的基本条件：一是随着高速公路网络的不断完善和港珠澳大桥的规划建设，我市交通条件显著改善，成为港澳及珠三角通往粤西和大西南的交通枢纽，区位优势日益显现；二是ＣＥＰＡ以及“大珠三角”和“泛珠三角”战略的实施，使我市作为珠三角西部中心城市的战略地位更显重要；三是我市丰富的土地资源，扎实的产业基础，独特的侨乡优势，高素质的劳动力以及优良的生产生活生态环境，为承接发达国家和珠三角先发地区产业转移，利用外资民资加快发展提供了有利条件；四是我市人均ＧＤＰ超过２０００美元，已进入经济社会发展的快车道。更重要的是，当前我市政通人和，心齐气顺，全市上下加快发展的愿望非常强烈。只要我们因势利导，善于借鉴先发地区的经验教训，少走弯路，充分发挥我市的后发优势，坚持科学发展观，促进经济社会全面、协调、可持续发展，就一定能开创我市现代化建设的新局面。

　　２００４年政府工作的指导思想是：以邓小平理论和“三个代表”重要思想为指导，以科学发展观为根本指针，全面贯彻落实十六大、十六届三中全会、中央经济工作会议和省委九届四次全会、市委十届二次全会及张德江书记视察我市重要讲话精神，继续推进工业强市、民资外资富市、科教兴市、环境优市战略的实施，实现经济发展速度与质量、效益相统一，经济建设与社会发展相统一，城市发展与农村发展相统一，增加投入与提高人民生活水平相统一，为完成“十五”计划，建设全面、协调、可持续发展的现代化大城市奠定坚实基础。

　　贯彻上述指导思想，我们必须以科学发展观为主线，努力实现“六个争先”：一是以抓好高速公路、电厂、港口航道建设为重点，努力实现基础设施建设争先；二是以发展高新技术产业、优势制造业、现代物流业、旅游业为重点，发挥“榕树效应”，实施名牌战略，努力实现产业发展争先；三是加大招商引资力度，创新方式方法，内外源并举，努力实现招商引资、发展民营经济争先；四是以“三化”解决“三农”问题为重点，努力实现城镇化建设争先；五是以发掘侨乡文化底蕴，打造侨乡品牌为重点，努力实现文化名市建设争先；六是以转变观念、制度创新、科学决策、以人为本为重点，建设高效廉洁的民本政府，努力实现政务环境建设争先。

　　今年我市经济社会发展的主要预期目标是：全市生产总值增长１１％，地方财政一般预算收入增长１２％，居民消费价格指数稳中略升，城镇登记失业率控制在３．５％以下，人口自然增长率控制在６．２３‰以下，城镇在岗职工年人均工资增长７％，农村居民年人均纯收入增长４％。

　　我们要着力抓好以下工作：

　　一、 加强产业建设，提高综合竞争力

　　加快发展高新技术产业。要找准我市发展高新技术产业的突破口，实施错位发展，在电子信息、新材料、生物制药和光机电一体化等领域加快推进，特别是要充分发挥政策的导向作用，重点发展电子元器件、电子外部设备和新兴信息技术，把我市打造成为全省乃至全国打印机和线路板的重要生产基地，尽快改变我市电子信息产业落后的局面。进一步加大火炬计划、重点产品计划、科技成果推广计划的实施力度，加快高新技术产业化进程。继续推进“５ｅ”工程。发挥政府科技顾问的作用，培育以“生产力促进中心”为依托的科技中介服务机构。加大企业技术创新中心、民营科技园区的建设力度，推动高新技术企业与民营科技企业快速发展，增强产业技术创新能力。

　　打造优势制造业“航母”。推进重点产业基地建设，壮大产业集群。要把纸业、摩托车及零配件、化纤、集装箱、五金卫浴等打造成为全国规模一流的产业基地，把不锈钢制品、纺织服装、平板玻璃、铝材、食品、电池、制鞋、电声器材等打造成为省内规模一流的产业基地。大力培育和扶持发展石油重化工、汽车配件、化学制品和珠宝加工等产业。推进亚太纸业、大长江扩产、维达扩建、名冠、益胜浮玻二线、华尔润、华泰、依利安达五期、潮连钻石岛等一批重大工业项目建设。继续办好机电产品博览会、水暖卫浴设备展销会、不锈钢制品展销会、牛仔服装节。大力实施名牌战略，增创国家、省级名牌产品和驰名商标，提升我市制造业的竞争力。

　　大力发展现代物流业和服务业。抓住ＣＥＰＡ实施的机遇，加强与港澳在现代物流业、金融业、旅游业、会展业和中介服务业上的合作交流。以新会港、台山广海港、高沙码头、外海外贸码头为依托，建设现代物流基地。近期积极做好香港大昌行在新会港建立进口农产品物流中心和保税仓的相关工作。加快发展旅游业，加大宣传推介力度，进一步打造温泉、沙滩、人文、生态等品牌，办好２００４中国（江门）侨乡旅游节。抓好开平碉楼申报世界文化遗产工作。发展假日经济、旅游经济和会展经济，培育和扩大住房、汽车、通讯、教育、文化、医疗、体育健身和旅游休闲等消费热点，推动消费结构升级。

　　二、增创招商引资新优势，发展壮大民营经济

　　加大招商引资力度。创新方式方法，引入现代企业营销理念，抓好招商引资工作。一方面要瞄准世界５００强，设立项目专责小组，组织小分队“敲门招商”；另一方面要通过建立中小企业工业园等方式，吸引中小企业的集聚。继续发挥政协委员、海外社团和行业协会（商会）等的桥梁作用。运用产业招商、中介招商、代理招商、网络招商、以侨引商、以商引商等多种方式，拓宽招商引资渠道。建立招商引资工作层级责任制，把投资项目跟踪服务工作落实到人。要重视招商引资奖励政策的兑现。积极鼓励外资、民资企业增资扩产。

　　提高园区建设水平。按照“有效益、有特色、可持续发展”的思路，统一规划，优化整合全市工业园区资源。鼓励外资民资开发建设上规模、上水平的工业园区。完善工业园区配套设施，提升承载能力和接受辐射能力。进一步优化园区软环境，设立综合性的投资服务机构，积极导入ＩＳＯ质量管理体系，创新管理方式。市区重点抓好高新技术产业开发区、江沙工业走廊、银洲湖区域３个工业园区，各市集中抓好１－２个核心工业园的建设，为吸引产业集聚，扩展产业链条，发展园区经济起示范作用。

　　努力扩大出口。积极贯彻国家调整出口退税的政策措施，做好新形势下外贸出口工作。推进外贸主体和出口市场多元化，鼓励支持民营企业自营出口、扩大出口。大力实施科技兴贸，优化出口商品结构，提高出口效益。加快“走出去”步伐，推动我市有实力的企业开展境外加工装配业务，利用国际资源加快发展。

　　继续大力发展民营经济。全面落实民营经济发展政策。引导民营企业制度创新、技术创新和管理创新。鼓励发展科技型、出口创汇型、就业型和农副产品深加工型企业，促进民营企业做大做强，今年民营经济占全市工业总产值和出口总值的比重争取达３５％和２５％。进一步健全民企服务体系，切实帮助民企解决管理、融资、信息、技术、人才等方面的问题，推动民营经济发展上新台阶。

　　三、以增加农民收入为核心，壮大县域经济

　　全力推进县域经济加快发展。以工业化为龙头，以城镇化为载体，以农业产业化为依托，以科技进步为动力，完善农村经济服务体系，发展专业镇，培育特色经济，加快贫困村脱贫步伐，推动镇村经济较快增长，实现增强县域经济实力，提高农民生活水平的目标。

　　加速农业产业化进程。认真落实今年中央一号文件精神，促进农业增效，农民增收。继续推进农业结构调整，优化农业区域布局，稳定２８１万亩基本农田，积极发展“三高”农业和生态农业。进一步完善农业社会化服务体系，活跃各种农业专业化组织，提高农业产业化水平。重点发展无公害农产品，力争年内５０％的镇建立无公害农产品生产基地。推进农业信息网建设，加快农业新技术、新品种的推广。形成农产品种养、加工、流通“一条龙”，增加农产品附加值。优化林业结构，促进林业产业发展。改善以水利、公路、电网为重点的农村基础设施。大力发展海洋经济，提高海洋渔业、临海工业、海洋运输业和滨海旅游业的发展水平。

　　加快城镇化进程。以中心镇建设为重点，科学制订规划，继续抓好并镇并村工作。新会、蓬江、江海三区要按省定的标准，今年内把符合条件的镇、村，基本完成改为办事处和社区居民委员会的工作，其他各市也要做好这项工作。增强城镇吸纳农村人口、安置农业富余劳动力就业的能力。抓好规范农民建房的试点和推广工作。

　　深化农村改革，减轻农民负担。健全减轻农民负担责任制和监督机制，巩固农村税费改革成果。涉农收费一律公开；继续抓好农村中小学乱收费和农民建房乱收费等专项治理，防止农民负担反弹。按照省政府的部署，加快取消农业税的步子。深化农村土地制度改革，积极推进农村集体建设用地使用权流转；保障农民土地承包合法权益，鼓励农民在承包期内依法、自愿、有偿流转土地承包权。加强对农民职业技能培训和就业培训，为贫困村和被征地农民免费进行就业培训，提高就业能力。深化镇级财政管理体制改革，建立激励型财政体制，推广市区财政收入增长分成的做法，理顺各市（区）对镇（办事处）的财政管理体制。

　　四、加快体制创新，努力实现改革目标

　　深化国有企业和公有资产管理体制改革。加大力度，以甘化集团、中旅集团、汽运集团、浮玻集团、农药厂等企业改制为重点，争取年内全面完成市直国有企业改革目标。切实做好改制、重组、破产国有企业职工安置工作。建立国有资产经营预算制度和经营业绩考核体系，积极探索国有资产经营、监管的有效形式。做大做强市公用设施投资控股公司、市基础设施投资控股公司等企业。建立行政事业资产流动制度，加强对闲置资产的管理和调度，盘活国有资产，提高资产运营效益。加强市产权交易中心建设，规范国有资产交易行为。

　　推进投融资管理体制改革。确立企业的投资主体地位。全面推行社会投资基本建设项目登记备案制度。培育投资中介服务机构，建立民间担保中心。积极发展和利用资本市场，鼓励有条件的龙头企业、民营企业和高新技术企业改制上市。加快发展产权、信息、劳动力、房地产等要素市场。继续整顿和规范市场经济秩序。推进信用体系建设，营造良好的社会信用环境。

　　建立健全公共财政体系。大力培育地方税源，严格规范非税收入管理，确保财政收入持续稳定增长，提高地方财政收入占ＧＤＰ的比重。强化部门预算、国库集中收付、政府采购和“收支两条线”管理。把社会保障、教育、卫生、文化等公共事业作为财政支出安排的重点。建立财政支出绩效评价体系，提高财政资金使用效益。建立财政抗风险应急预警机制，继续做好防范和化解地方金融风险工作，妥善清理政府债务。

　　五、加快基础设施建设，实施可持续发展战略

　　抓好交通能源基础设施建设。要为台山电厂一期１、２号机组满负荷发电，３、４、５号机组加快建设，二期工程早日上马创造必要条件和提供大力支持，做好崖门电厂筹建的前期工作，努力把我市建成全省重要的能源基地。加快推进江珠、江中、江鹤二期高速公路建设，做好江肇高速、广珠轻轨江门支线筹建的前期工作，争取在二、三年内，实现我市与港澳和珠三角其它地区一个半小时经济生活圈。加快广海港、新会港二期建设和崖门航道疏浚，推动临港经济的崛起。

　　高起点、高标准做好城市总体规划和可持续发展规划。 《江门市城市总体规划》（修编）要在年内提交市人大常委会审议；滨江新区规划实行国际招标；按照“一流的规划单位，一流的研究成果”的要求，组织制定全市可持续发展规划。加大规划实施力度，精心组织市区三大组团规划的实施，抓好滨江大道、东海路至南环路、江海路二期、江礼大桥、会展中心、五星级酒店、垃圾处理场等城市重点项目的建设，进一步改善江沙沿线交通和景观。经营好土地、交通、环境、信息、公用设施等城市资源，实现城市建设的良性循环和滚动发展。

　　加大环保工作力度，促进人与自然和谐发展。坚持“既要金山银山，又要绿水青山”，大环境建设与小环境配套有机结合，在现有２４３万亩公益生态林的基础上进一步扩大造林面积，在城镇建成区营造点（小区）、线（道路）、面（公园、公共绿地）结合的无处不绿、处处有树的绿化环境，形成“城在林中，城中有林”的生态格局。大力推进江门河、天沙河、会城河、紫水河水环境综合整治工程，狠抓污染源治理，加快各市、区和中心镇生活污水处理厂的建设，确保西江、潭江两大干流江门段水质不加重污染。采取有效措施整治机动车尾气和噪声污染。巩固工业污染源整治达标排放成果，严防和及时遏制反弹。完善环保管理机制，落实年度环境保护责任制和各项环境整治任务，确保创建国家环保模范城目标的实现。建立和完善森林、气象、地震、地质、海洋等自然灾害监测预警机制，保护生态文明和社会文明。

　　六、建设有侨乡特色的文化名市，发展社会各项事业

　　加快发展具有侨乡特色的文化教育事业。大力弘扬“开拓创新、文明开放、勤劳创业、团结奉献”的侨乡人精神。以创建文明城市为契机，深入开展群众性精神文明创建活动。办好首届“中国（江门）侨乡华人嘉年华”，使其成为发挥侨乡新优势，建设文化产业，打造旅游品牌的有效载体。同时，做好第五批荣誉市民的授荣工作。继续优先发展教育事业。推进教育管理体制和办学体制创新，鼓励和规范社会力量办学，整合教育资源。在普及提高九年义务教育的基础上，加快发展高中教育和职业教育，进一步办好五邑大学等高校。搞好江门职业技术学院、一中新校区、技工学校扩建的建设。抓紧完成江南小学、启明小学的改薄工作。扶持农村教育，切实解决失学儿童问题。抓好市直幼儿园的改革工作。高质量建设好美术馆、华侨博物馆、电视中心、名人馆和星光大道等重点文化工程。搞好以五邑侨乡为题材的歌曲、戏剧、小说、画作、电视片的创作评选活动。加强社区文化建设。保护、挖掘、开发民间文化资源。鼓励多渠道投资兴办文化产业，扶持文化龙头企业。创新文化管理体制，深化文化企事业单位改革。

　　大力实施人才强市战略。重点围绕公务员队伍、专业技术人员队伍、企业经营管理人员队伍的建设，在培养、引进、用好人才等环节上下功夫。深化人事制度改革，营造人才集聚和发挥才干的环境，畅通人才流通渠道。抓好人才市场和“江门人才网”建设。

　　协调促进社会各项事业全面发展。发展妇女儿童事业。加强城市社区建设。加快釜山公园二期、体育公园、体育综合训练馆等体育设施建设，广泛开展全民健身活动。推进双拥共建和优抚安置工作。抓好全民国防教育，提高国防动员建设和民兵预备役工作水平。完善防空设施建设管理。继续做好外事、侨务、台务、残联、宗教、老龄、档案等工作，促进社会各项事业蓬勃发展。

　　七、加强公共服务工作，提高社会保障水平

　　完善公共卫生体系。做好非典、禽流感和其他传染病的预防和控制工作。进一步健全疾病预防控制体系、卫生执法监督体系及医疗救治体系，完善应急处理机制，提高对突发公共卫生事件的处理能力。加快市疾病预防控制中心建设。积极推进医疗卫生改革，整顿医疗乱收费。打击取缔无证行医，规范医疗市场秩序。加强乡镇卫生院和三级医疗预防保健网络建设，优化组合农村医疗网点。加强农村医务人员培训，提高农村预防保健和基本医疗服务水平。大力推进新型农村合作医疗，全市新型农村合作医疗人口覆盖率年内要达到６０％。普及公共卫生和健康宣传教育，引导广大市民养成健康文明的公共卫生和生活习惯。建立国家卫生城市长效管理机制，持久深入地开展全民爱国卫生运动，提高全社会公共卫生水平。

　　做好就业和再就业工作。全面落实就业与再就业各项政策措施，推进城乡统筹就业。加大就业岗位开发力度，全年新增１．６万个就业岗位。强化职业技能培训和创业培训，完善就业服务体系，协调提供小额就业担保贷款。对“４０５０”就业困难人员实施就业援助。切实做好高校毕业生就业工作。加强劳动监察和劳动关系协调，保障劳动者合法权益。

　　健全社会保障体系。加快社会保险市级统筹步伐，推进全民社保计划。重点抓好个体私营等非公有制企业的参保工作，做好下岗职工基本生活保障与失业保险并轨的后续工作。逐步提高城市居民最低生活保障水平，扩大城镇职工基本医疗保险覆盖面。强化社会保险基金征缴和监管。对农村年人均收入１５６０元以下的困难群众全面实行低保。落实为被征地农民购买养老保险的工作。

　　切实关心人民群众生活。继续抓好“十项民心工程”建设。根据财力，适当提高公务员和教师的收入水平。切实帮助困难群众解决子女上学、就业、就医、住房、打官司等方面遇到的问题。在历年解决集体年收入３万元以下的村委会和人均纯收入１５００元以下的农民脱贫工作取得显著成绩的基础上，今年要实现５０条村脱贫的目标，争取用三年左右时间，基本解决贫困村脱贫的问题。抓好城乡水利防灾减灾工程、农村饮水工程和以解决水库移民住房为重点的农村安居工程建设。落实人口与计划生育法等法律法规，稳定低生育水平，提高出生人口素质。做好救助管理和救灾救济工作。加强信访工作，认真解决人民群众反映强烈的社会热点和难点问题。

　　认真抓好安全生产。加强安全生产监督管理，落实安全生产责任制。加强重点行业、重点企业的安全生产监管，推行注册安全主任制度，严防重特大安全事故发生。

　　切实维护社会稳定。严密防范和严厉打击境内外敌对势力、暴力恐怖势力和“法轮功”邪教组织的破坏活动。坚持“打防结合、预防为主”的方针，坚决打击各类违法犯罪活动。加强监狱、劳教工作。突出抓好“禁毒０３工程”。积极推进安全文明社区、“无毒”社区的建设，净化社会环境。加强社会治安综合治理。积极疏导和化解人民内部矛盾，维护社会大局稳定。

　　八、提高行政能力，建设民本政府

　　加强政府作风建设和廉政建设。为适应现代化建设加快发展的需要，今年机关作风建设要进一步加大力度，要以建设学习型、服务型、法制型、实干型、廉洁型政府为目标，以营造交通、政务、人才、法制和生活“五好”环境为基本要求，倡导“亲民、为民、务实、清廉”作风，并从制度、措施上狠抓落实。一是简政放权，管理重心下移，进一步转变政府职能，搞好市县政府机构改革。二是实施管理创新，在试点的基础上，有步骤地在市直机关导入ＩＳＯ质量管理体系。三是重点抓好经营性土地使用权招标拍卖、建设工程项目公开招投标、政府采购、产权交易进入市场等制度的落实，从源头上防止腐败。四是保持政府与人民群众联系渠道的畅通，自觉接受人民群众监督。完善政府为群众、为企业服务的平台，及时解决企业、基层与群众反映的热点难点问题。采取有效措施，进一步解决“文山会海”，提高行政效率。厉行节约，反对奢侈浪费。

　　推进依法行政和民主法制建设。自觉接受人大及其常委会的监督，切实办好人大代表议案、建议和政协提案。主动听取各民主党派、工商联、无党派人士及各人民团体的意见和建议。坚持科学民主决策，建立和完善重大问题集体决策制度、民主协商制度、专家咨询制度、社会公示和听证制度。发展电子政务，办好“五邑网上论坛”，健全新闻发布会制度，提高决策透明度和公众参与度。全面推进政务公开、厂务公开、村务公开，完善村民自治和城市社区居民自治，健全职工代表大会和其他形式的企事业民主管理制度，维护人民群众当家作主的权利。认真贯彻《行政许可法》，规范行政执法行为，提高依法行政水平。加强行政执法监督，完善行政监督制度，做到有权必有责、用权受监督、侵权要赔偿。

　　各位代表，我们处在一个关键的历史阶段，我们所面临的任务光荣而艰巨。让我们同心同德，精诚团结，全面贯彻“三个代表”重要思想，牢固树立和落实科学发展观，在中共江门市委的正确领导下，抢抓机遇、开拓进取，为把我市建设成为全面、协调、可持续发展的现代化大城市而努力奋斗！
